# Healing and functional outcomes after treatment of high anal fistula with a modified anal fistula plug: a prospective cohort study

**DOI:** 10.3389/fmed.2026.1767824

**Published:** 2026-04-09

**Authors:** Rui Zhang, Hanlin Gong

**Affiliations:** Department of Integrated Traditional Chinese and Western Medicine, West China Hospital of Sichuan University, Chengdu, Sichuan, China

**Keywords:** anal fistula, anal fistula plug, postoperative pain, sphincter preservation, time to healing

## Abstract

**Objective:**

To evaluate whether a modified anal fistula plug combined with a drainage seton can improve postoperative pain and 12-month healing rates, and to assess its effects on healing time, recurrence, bowel-function recovery, and other outcomes.

**Methods:**

We conducted a single-center comparative cohort study at West China Hospital, including 140 patients with cryptoglandular anal fistula treated between January 2022 and December 2024. Patients were categorized by whether they received the anal fistula plug group (AFP) or not. Both groups followed our institution-specific postoperative pathway combining Chinese herbal fumigation with a protocolized recovery bundle.

**Results:**

Baseline clinical characteristics were comparable between groups. Compared with contrast group, the AFP group showed faster postoperative recovery, with lower early postoperative pain and a shorter median time to healing. Functional recovery and overall clinical effectiveness were also higher early after surgery. At 12 months, overall effectiveness was similar between groups and recurrence did not differ significantly. Subgroup analysis suggested a greater acceleration of healing in patients with longer preoperative disease duration (≈45–365 days).

**Conclusion:**

The modified AFP with drainage seton facilitates early recovery, significantly reduces postoperative pain, and shortens time to healing without increasing costs or hospital stay. Over 12 months, long-term efficacy and safety are comparable to conventional treatment, supporting the plug as a sphincter-preserving, cost-conscious alternative.

## Introduction

Anal fistula is a chronic infectious condition characterized by an abnormal tract between the anal canal or rectum and the perianal skin. It predominantly affects young and middle-aged men, with an estimated incidence of 8–23 per 100,000 individuals (1). Based on the anatomical relationship between the fistulous tract and the sphincter complex, anal fistulas are generally classified into low and high types. High fistulas are primarily of cryptoglandular origin, arising from infection of the anal glands at the dentate line, which initially forms an abscess and subsequently penetrates surrounding tissues under persistent inflammation, ultimately developing into a chronic fistulous tract. A smaller proportion of cases are secondary in nature and may result from Crohn’s disease (2), tuberculosis, trauma, radiation therapy, hidradenitis suppurativa, HIV and other sexually transmitted infections, or malignancies of the anal canal or rectum.

High anal fistulas typically present with recurrent or persistent perianal discharge, suppuration, swelling, and pruritus (3). Owing to the dense distribution of sensory nerves in the perianal region, patients often experience marked pain together with substantial defecatory dysfunction. The disease is chronic and prone to recurrence, leading not only to physiological impairment but also to considerable psychological and social burden. From a clinical perspective, the diagnosis, long-term management, complex surgical interventions, and postoperative complications of high anal fistulas contribute to substantial direct medical costs. In addition, repeated disease flares and prolonged postoperative recovery result in work absenteeism and reduced productivity, further increasing the indirect economic burden on patients and the healthcare system. Consequently, the comprehensive management of high anal fistula represents not only a surgical challenge but also a significant public health concern.

Surgery remains the primary treatment for high anal fistula; however, its efficacy is often accompanied by a substantial risk of sphincter injury, which may lead to fecal incontinence (4) and significantly impair quality of life. Owing to the unique anatomy and physiological function of the anorectal region, postoperative wounds typically heal by secondary intention, resulting in a prolonged healing period and continuous exposure to intestinal flora, thereby increasing the risk of infection. Postoperative pain represents another major barrier to recovery. Previous studies have demonstrated that a large proportion of patients experience moderate pain shortly after anorectal surgery, which frequently escalates to severe pain during dressing changes; similar pain patterns are commonly observed after fistulotomy and seton placement (5). Such intense pain contributes to defecation anxiety, sleep disturbance, and marked reductions in overall quality of life (6).

Current surgical approaches struggle to balance healing efficacy with sphincter preservation. The ideal management of anal fistula should minimize sphincter damage, promote tract closure, preserve anorectal function, and prevent recurrence (7). With advances in the understanding of anal cushion anatomy and sphincter physiology, surgical concepts have shifted from radical excision toward functional preservation, emphasizing efficacy and safety while maintaining sphincter integrity (8). In recent years, biomaterial-based anal fistula plug technology has garnered increasing attention as a sphincter-preserving strategy. This technique occludes the internal opening without dividing the sphincter fibers, promoting fistula closure while minimizing functional impairment. As such, it has become one of the commonly utilized options for managing high anal fistulas. However, the clinical outcomes of this approach vary depending on the surgical technique employed. In several countries, including Germany, it was once common practice to combine the use of anal fistula plugs, which are metabolized by the body over time, with mucosal flap advancement for closure of the internal opening. However, this method yielded healing rates just slightly over 50%, with limited success, and has since been largely abandoned in favor of alternative techniques. We postulate that the suboptimal outcomes associated with traditional methods may be attributed to the closed management of the fistula tract and the reliance on the passive absorption of the plug material. In contrast, the present study adopts a different therapeutic approach: the use of a loose seton to maintain the patency of the fistulous tract, thereby facilitating gradual healing independent of the plug’s absorption, resulting in superior healing outcomes. To further standardize and optimize this procedure, we have developed a single-use device for the management of the internal opening and proximal tract. This instrument enables controlled debridement and de-epithelialization of the internal opening while providing stable fixation. The anal fistula plug then serves as an absorbable scaffold to seal the internal opening. When combined with the routine use of a draining loose seton, this method ensures continuous drainage, reduces the risk of postoperative infection, and may lower early failure rates while minimizing injury to the sphincter complex.

While previous studies suggest potential benefits of Anal Fistula Plug (AFP), their small sample sizes raise concerns about bias, precluding definitive conclusions on its efficacy and safety. The current evidence focuses predominantly on short-term healing rates, with scarce data on long-term recurrence and anal function (9). Furthermore, its real-world effectiveness, cost-effectiveness ratio, and time to complete healing require comparative evaluation against conventional treatments.

To address the lack of high-quality evidence regarding long-term efficacy and recurrence in sphincter-preserving treatments for high and complex anal fistulas, we conducted a prospective study with extended follow-up. Using a self-developed internal-opening management device combined with a draining loose seton, and comparing it with conventional seton therapy, we systematically evaluated postoperative pain, healing, anorectal function, and recurrence. By enrolling consecutive patients and applying propensity score weighting to control for confounding, this study aimed to reflect real-world clinical practice and provide stronger evidence to guide treatment decisions and improve long-term quality of life in affected patients.

## Methods and materials

### Study design and ethics

This single-center, prospective cohort study was conducted at West China Hospital, Sichuan University. The study protocol was reviewed and approved by the Ethics Committee of West China Hospital, Sichuan University Approval No.2023(2446). All procedures involving human participants were performed in accordance with the ethical standards of research committee and with the Declaration of Helsinki and relevant guidelines and regulations. Written informed consent was obtained from all participants prior to their enrollment in the study.

### Participants and setting

Due to the pragmatic study design, treatment allocation was non-randomized. From January 2022 to December 2024, a total of 140 consecutive eligible patients undergoing surgery for high anal fistula were enrolled from the Department of Integrated Traditional Chinese and Western Medicine at West China Hospital. The choice of procedure was determined through shared decision-making between the surgeon and the patient, based on fistula anatomy, feasibility of plug placement, patient preference, and continence preservation. Seventy-three patients who received treatment with a self-modified anal fistula plug combined with a drainage seton were assigned to the Plug Cohort, and 67 patients who underwent the classical cutting-seton procedure were assigned to the Contrast Cohort. As both procedures were routinely performed during the recruitment period, the cohort sizes were approximately balanced without quota-based assignment. Baseline characteristics were compared and adjusted in subsequent analyses to reduce confounding.

#### Inclusion criteria

(1) Diagnosed with high anal fistula according to the 2006 “Clinical Guidelines for the Diagnosis and Treatment of Anal Fistula” (i.e., fistula tract courses above the deep layer of the external sphincter); (2) Aged 18–65 years; (3) Initial onset of high anal fistula and first hospitalization; (4) American Society of Anesthesiologists physical status I-II with stable vital signs; (5) Normal anal morphology and function, with no previous history of anal surgery; and (6) Agreement to undergo general anesthesia and one-stage radical surgery, and commitment to postoperative follow-up.

#### Exclusion criteria

(1) Acute stage of infection (defined as presence of systemic symptoms like fever, or local abscess requiring emergency incision and drainage); (2) Comorbid with other anorectal diseases such as anal fissure, Crohn’s disease, tuberculous fistula, anorectal tumors, inflammatory bowel disease, acute/chronic diarrhea, or perianal eczema; (3) Uncontrolled severe primary diseases of the cardiovascular, cerebrovascular, hepatic, or renal systems, diabetes mellitus (preoperative fasting blood glucose >8.0 mmol/L), or infectious diseases; (4) Pregnant, lactating, or menstruating female patients; and (5) Concurrent participation in other clinical trials that might interfere with the outcomes of this study.

### Exposure and cohorts

Exposure of interest was surgical procedure type. The exposed cohort underwent treatment with a modified anal fistula plug based on the patented Fistula Orifice Sealing and Draining Device (Chinese Patent No. ZL 2019 1 1157806.0) in combination with a drainage seton, which served as the core implant for internal orifice closure. The non-exposed cohort underwent the classical cutting seton procedure alone. In contrast to traditional treatments such as the fistula plug method used in some countries, our method uses a loose seton, leaving the fistula tract open for drainage until secretion stops. This approach avoids the issues associated with plug metabolism and leads to faster recovery and significantly better healing rates. Postoperative analgesia was provided according to the institutional protocol. Rescue analgesics were administered when patients reported inadequate pain control, and related use was documented in the medical record.

### Data collection and variable definition

Postoperative follow-up was conducted by study investigators through a combination of clinic visits and telephone contacts, using standardized case report forms. Wound care and dressing changes were performed in the outpatient clinic according to established colorectal practice, tailored to individual wound conditions. A routine postoperative visit was scheduled at 1 month. Additional visits were arranged if clinical indications arose, such as persistent drainage, local induration, pain, or suspected infection. During each follow-up contact, pre-specified variables were collected in a uniform manner.

#### Baseline variables

Age, sex, body mass index, disease duration, comorbidities, fistula type (simple/complex).

#### Outcome variables

##### Primary outcomes

Pain was assessed on postoperative days 0, 1, 7,10 and 14 using the Visual Analogue Scale (0–10, with higher scores indicating greater pain). Healing rate at 12 months was determined at the 12-month follow-up. Healing was defined as the absence of pus discharge, closure of the external opening, no tenderness or induration on digital rectal examination, and confirmed fistula tract obliteration on perianal ultrasound or MRI.

##### Secondary outcomes

Perioperative outcomes included operative time, total hospitalization cost, and postoperative length of stay. Wound-healing time was defined as the number of days from surgery to complete epithelialization without further dressing requirements. Early postoperative complications included incision edema, fever, urinary retention, and bleeding. Recurrence was defined as the reappearance of fistula symptoms at the original surgical site after confirmed healing and was assessed at 24 months. Safety was evaluated by documenting all serious and device-related adverse events and grading overall safety on a four-level scale.

Clinical healing was determined by the investigators during outpatient evaluations. Time to healing was defined as the earliest date when the predefined healing criteria were met based on local examination and clinical documentation. When clinical findings were ambiguous or recurrence was suspected, imaging (e.g., endoanal ultrasound or MRI) was performed per routine protocol.

### Sample size

We planned to enroll approximately 140 patients with high anal fistula during the recruitment period from January 2022 to December 2024. This sample size was estimated based on the feasible surgical volume at our center. With 73 patients in the anal fistula plug cohort and 67 in the contrast cohort, the study has 80% power at a two-sided alpha of 0.05 to detect a clinically important difference of approximately 25% in healing rate. The total study duration extended to December 2024 to ensure a minimum of 2-years follow-up for all participants.

### Statistical methods

**Figure 1:**
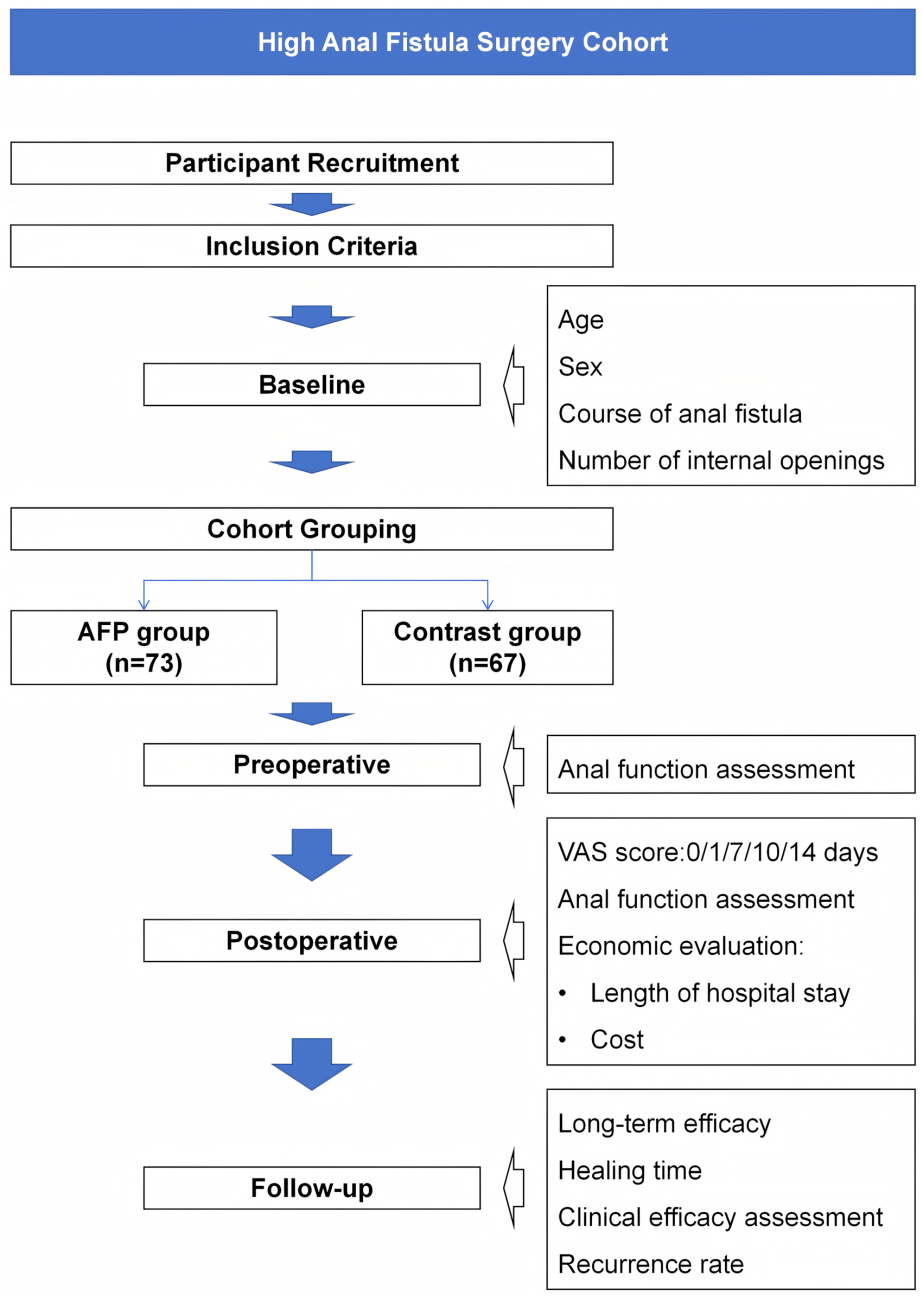
Flowchart of the study design.

All statistical analyses will be conducted using SPSS Statistics 26.0. Continuous variables will be tested for normality using the Shapiro–Wilk test and presented as mean ± standard deviation or median (IQR), with comparisons made using the independent-samples *t*-test or Mann–Whitney U test, respectively. Categorical variables will be reported as numbers (percentages) and compared using the Chi-square test or Fisher’s exact test. To assess the independent effect of the Kanloushun procedure, multivariable regression models will be applied: logistic regression for binary outcomes, linear regression for continuous outcomes, and Cox proportional hazards models for time-to-event outcomes. Subgroup analyses will be performed to examine the influence of baseline factors. All tests will be two-sided with statistical significance set at *p* < 0.05. For variables with <5% missing data, complete-case analysis will be used; otherwise, multiple imputation (five datasets) will be performed, and pooled estimates will be reported (Figure 1).

## Result

### Comparability of baseline demographics and clinical features

**Figure 2:**
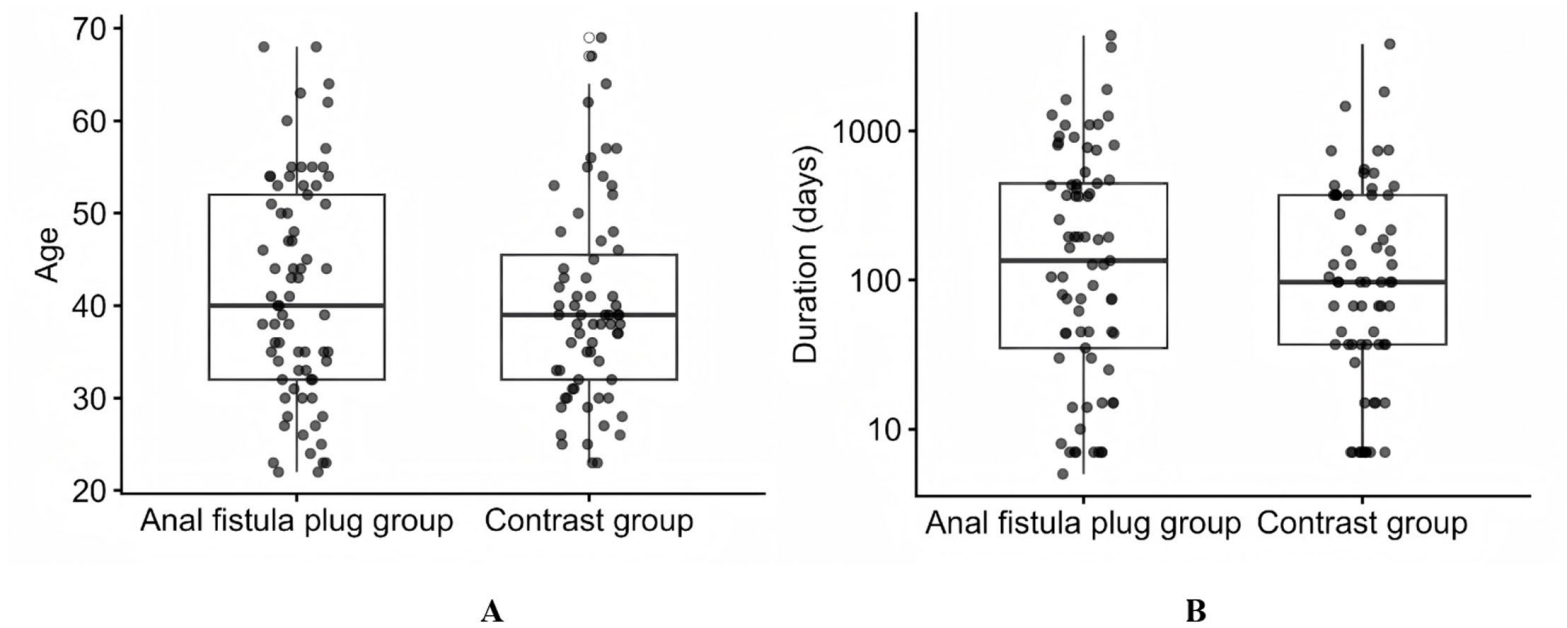
Baseline characteristics of the AFP group and the contrast group: **(A)** Boxplot of age distribution between the two groups; **(B)** Boxplot of disease duration between the two groups.

As shown in Table 1, a total of 140 participants were included (AFP group, *n* = 73; contrast group, *n* = 67). Baseline demographics and disease characteristics were well balanced between groups (Figure 2), indicating negligible baseline imbalance. Therefore, subsequent between-group comparisons are unlikely to be confounded by baseline differences.

**Table 1:** Baseline demographic and clinical characteristics of two groups.

Baseline characteristic	Anal fistula plug group	Contrast group	SMD
Age, years	40.00 (32.00–52.00)	39.00 (IQR 32.00–45.50)	0.08
Male, *n* (%)	61 (83.6)	58 (86.6)	−0.08
Female, *n* (%)	12 (16.4)	9 (13.4)	0.08
Duration, days	135.00 (35–446)	97.00 (37–372)	0.14

Data are presented as median (IQR) or *n* (%). SMD, standardized mean difference; IQR, interquartile range. An absolute SMD <0.10 indicates good balance between groups.

### Comparative trajectories of postoperative VAS pain across visits

**Figure 3:**
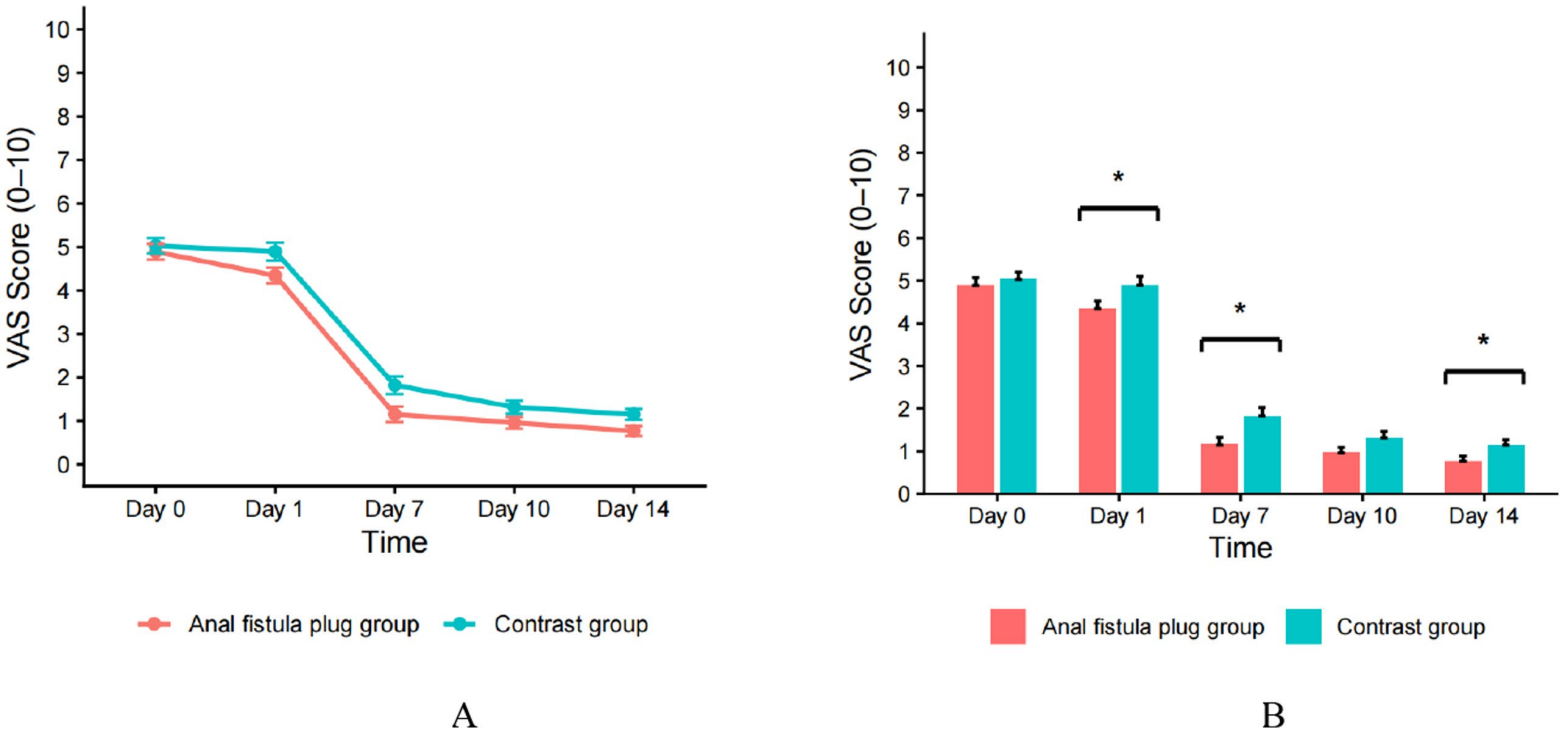
VAS pain in AFP and contrast groups. **(A)** Temporal changes in VAS scores for both groups; **(B)** Between-group comparisons of VAS scores at each follow-up time point.

VAS was assessed on POD 1, 7, 10, and 14. In both groups, VAS scores decreased over time. At baseline (preoperative), there was no between-group difference (*p* = 0.44). On postoperative day 1, day 7, and day 14, the AFP group had significantly lower VAS scores than the contrast group (*p* = 0.02, 0.005, and 0.01, respectively). On day 10, the difference approached but did not reach conventional statistical significance (*p* = 0.06). See Table 2 for details. Overall, the AFP group showed lower pain scores at several time points, suggesting a more favorable analgesic profile (Figure 3).

**Table 2:** Between-group comparison of VAS pain scores at baseline and postoperative follow-ups.

Group	Preoperative	Day 1	Day 7	Day 10	Day 14
Anal fistula plug group	4.89 ± 1.55	4.34 ± 1.55	1.15 ± 1.52	0.96 ± 1.16	0.77 ± 0.98
Contrast group	5.03 ± 1.44	4.9 ± 1.68	1.82 ± 1.66	1.31 ± 1.22	1.15 ± 1
*p*-value	0.44	0.02	0.005	0.06	0.01

### Change in bowel-function symptoms over follow-up by group

As shown in Table 3, a total of 73 participants were assigned to the AFP group and 67 to the contrast group. We compared preoperative baseline bowel-function outcomes between groups. The Pearson χ^2^ test without continuity correction was used when expected counts were adequate, and the Fisher’s exact test was used for small or zero counts. No significant between-group differences were observed at baseline, indicating comparable baseline characteristics.

**Table 3:** Baseline (preoperative) bowel-function symptoms.

Bowel-function assessment	Anal fistula plug (*n* = 73)	Contrast group (*n* = 67)	*p*-value	RD 95%CI
Urgency to defecate	11 (15.07%)	12 (17.91%)	0.65 (χ^2^ test)	−20.12–14.45
Need for pad or leak protection	3 (4.11%)	2 (2.99%)	1.00 (Fisher)	−8.84–10.58
Need for medication to control defecation	1 (1.37%)	0 (0.0%)	1.00 (Fisher)	−5.18–7.36
Impact on daily life	39 (53.43%)	27 (40.30%)	0.12 (χ^2^ test)	−10.16–35.02

At postoperative follow-up through the healing phase, all patients in both groups had complete resolution of the above bowel-function symptoms.

### Sustained efficacy and safety over extended follow-up

Clinical healing was assessed at 12 months postoperatively, and fistula recurrence was monitored up to 24 months. As shown in Table 4, using “cured + improved” as the definition of effectiveness, rates were 95.9% in the AFP group and 95.5% in the contrast group; the difference was not significant (Fisher’s exact test, *p* = 0.711). The risk ratio was 1.02 (95% CI, 0.94–1.10) and the odds ratio 1.46 (95% CI, 0.31–6.77).

**Table 4:** Comparison of healing status, relapse, and effectiveness between groups.

Outcome	Cured, *n*	Improved, *n*	Not healed, *n*	Relapse, *n*	Effective rate, %	Relapse rate, %
Anal fistula plug group (*n* = 73)	67	3	3	4	95.89%	5.48%
Contrast group (*n* = 67)	53	11	4	1	95.52%	1.49%
OR					1.46 (0.31–6.77)	3.83 (0.42–35.13)
RR					1.02 (0.94–1.10)	3.67 (0.42–32.03)
*P*-value					0.711	0.368

Relapse occurred in 5.5% of the AFP group and 1.5% of the contrast group, with no significant between-group difference (Fisher’s exact test, *p* = 0.368). The corresponding risk ratio was 3.67 (95% CI, 0.42–32.03) and the odds ratio 3.83 (95% CI, 0.42–35.13).

Safety outcomes were uniformly favorable. Both exudate and fever scores were 0 in each group, indicating good tolerability with no between-group differences.

### Group-wise comparison of time to clinical healing

**Figure 4:**
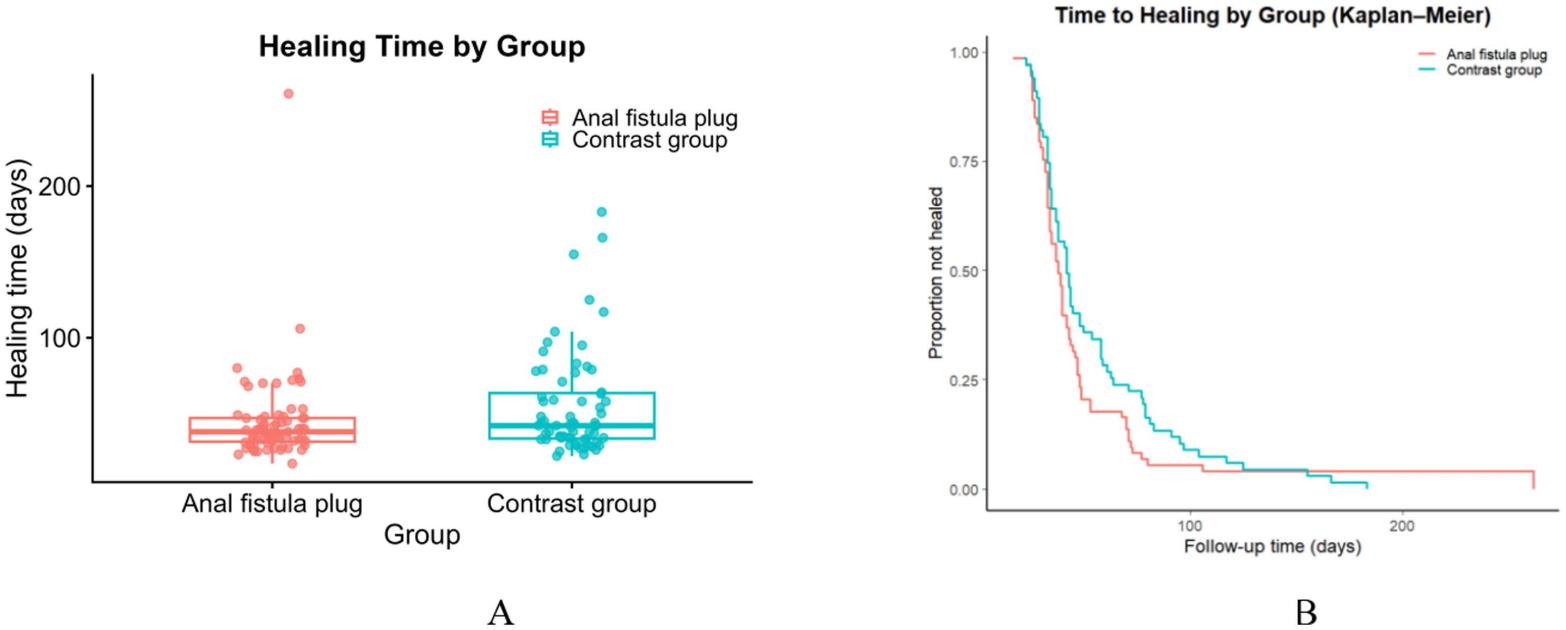
Healing outcomes in AFP and contrast groups: **(A)** Overall healing rates compared between groups; **(B)** Time-to-healing comparison between groups.

In the time-to-event analysis, 71 patients in the AFP group and 67 in the contrast group were included. As shown in Table 5, Kaplan–Meier curves showed faster healing in the AFP group (Figure 4B). The median time to healing was 38 days (95% CI, 34–40) in the AFP group versus 42 days (95% CI, 38–48) in contrast group (Figure 4A), yielding a statistically significant between-group difference (log-rank *p* = 0.0399). These findings indicate a modest but significant reduction in time to healing with use of the AFP.

**Table 5:** Time to healing by two groups.

Group	Median	95%CI	*P*-value
Anal fistula plug group (*n* = 71)	38	34–40	0.0399
Contrast group (*n* = 67)	42	38–48	

### Cost-effectiveness between the two groups

Inpatient resource use was similar between the two groups. As shown in Table 6, length of stay did not differ (3.69 ± 1.57 vs. 3.84 ± 2.64 days; Mann–Whitney U, *p* = 0.557). Total hospitalization cost was also comparable (¥9,445.7 ± 1,027.7 vs. ¥9,594.9 ± 1,573.9; *p* = 0.995). These findings indicate no additional inpatient cost burden associated with the intervention within the study period.

**Table 6:** Between-group comparison of inpatient stay and cost.

Group	Case group	Contrast group	*P*-value
Length of stay	3.69 ± 1.57	3.83 ± 2.64	0.556707
Hospitalization cost	9445.74 ± 1027.7	9594.95 ± 1573.9	0.994952

### Associations with healing time by strata of disease duration and fistula number

**Figure 5:**
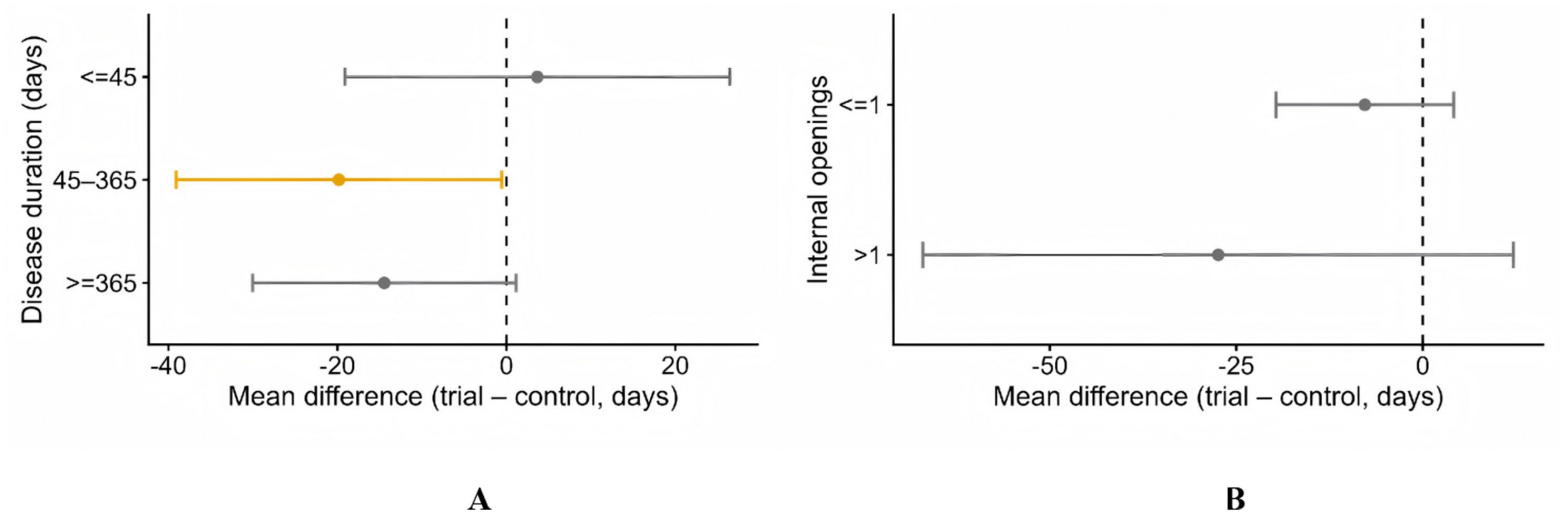
Correlates of time-to-healing: **(A)** Associations stratified by disease duration; **(B)** Associations stratified by internal opening.

We compared time to healing days between the AFP group and contrast group using stratified analyses. As shown in Table 7, in the 45–365 days stratum, the AFP group showed a statistically significant reduction in time to healing compared with contrast (MD = −21.19 days; 95% CI − 40.32 to −2.07). No significant difference was observed in the ≤45 days stratum (MD = 3.66 days; 95% CI − 19.13 to 26.44). In the ≥365 days stratum, the direction favored AFP group but did not reach statistical significance (MD = −13.60 days; 95% CI − 29.33 to 2.14) (Figure 5A).

**Table 7:** Number of internal openings—the AFP group vs. contrast group (outcome: time to healing, days).

Stratum	Mean difference	95% CI (lower)	95% CI (upper)	Conclusion
≤1	−7.76	−19.67	4.14	No significant difference between groups
>1	−27.42	−67.01	12.18	No significant difference between groups

As shown in Table 8, in the >1 openings stratum, the AFP group tended to heal faster without statistical significance (MD = −27.42 days; 95% CI − 67.01 to 12.18). A similar non-significant trend was seen for ≤1 opening (MD = −7.76 days; 95% CI − 19.67 to 4.14) (Figure 5B).

**Table 8:** Disease duration (days)—the AFP group vs. contrast group (outcome: time to healing, days).

Stratum	Mean difference	95% CI (lower)	95% CI (upper)	Conclusion
≤45	3.66	−19.13	26.44	No significant difference between groups
45–365	−21.19	−40.32	−2.07	the anal fistula plug group vs. contrast group faster; statistically significant
≥365	−13.60	−29.33	2.14	No significant difference between groups

MD = the anal fistula plug group − the contrast group. Negative MD indicates shorter time to healing in the AFP group. Statistical significance is determined by whether the 95% CI excludes 0.

## Discussion

This prospective cohort study demonstrates that both the AFP procedure and the traditional cutting seton technique are effective and safe strategies for managing high anal fistula, with comparable overall efficacy and safety profiles. However, the AFP procedure offers distinct advantages in facilitating early postoperative recovery. Specifically, patients in the plug cohort experienced significantly lower pain levels in the immediate postoperative period and achieved a markedly shorter median healing time compared to the contrast group. These findings underscore the clinical value of the plug technique in enhancing short-term quality of life and accelerating wound repair. Subgroup analyses further suggested that the healing acceleration benefit might be more pronounced in patients with a longer disease duration (45–365 days). Importantly, these recovery benefits were achieved without significantly increasing hospitalization costs or length of stay, indicating favorable cost-effectiveness. Regarding long-term outcomes and safety, no statistically significant differences were observed between the two groups in the 12-month overall efficacy rate (95.9% vs. 95.5%) or recurrence rate. Although the numerical recurrence rate was slightly higher in the plug cohort (5.5% vs. 1.5%), this difference was not statistically significant, and its clinical relevance warrants further investigation through larger, long-term studies. Both procedures exhibited excellent safety and tolerability. During follow-up through the healing phase, bowel function symptoms progressively improved in both cohorts. Specifically, the resolution of urgency may be attributed to the reduction of chronic local irritation and inflammation following fistula closure, which led to decreased persistent discharge, diminished defecation-related discomfort, and less sphincter spasm. In summary, the AFP minimally invasive technique serves as an effective alternative. While maintaining comparable long-term efficacy and safety to the traditional seton method, it provides significant benefits in reducing postoperative pain and accelerating healing without imposing an additional economic burden. It presents an attractive treatment option, particularly for patients with moderate disease duration who prioritize faster recovery and an improved perioperative experience.

Anal sphincter function is essential to postoperative quality of life, prompting growing preference for sphincter-preserving surgical approaches. The cutting seton facilitates secondary healing (10) through gradual sphincter division induced by sustained tension and chronic inflammation, which often results in greater postoperative pain and prolonged wound healing (11).

Recent systematic reviews highlight that sphincter-preserving and minimally invasive procedures such as LIFT, VAAFT, and AFPs reduce tissue trauma and avoid continuous sphincter division, thereby lowering postoperative pain, accelerating recovery, and improving quality of life (12). AFP typically requires only a small incision at the internal opening, followed by gentle tract cleaning and plug placement without enlarging the tract or dividing sphincter fibers, while maintaining drainage at the external opening. Consequently, AFP minimizes wound size, prevents tension-related damage, and better preserves sphincter function (13). Prior studies have also demonstrated its simplicity, low postoperative pain, minimal functional impairment, and extremely low incidence of incontinence (14) during follow-up.

In this study, the AFP group demonstrated shorter wound-healing time while maintaining satisfactory anorectal function, supporting the biological rationale that plug materials facilitate more orderly primary healing, whereas seton treatment results in secondary healing through gradual tissue division (15). In a systematic review by Garg (16), healing rates for AFPs ranged from 24 to 92% across 12 included studies, with low complication and infection rates, indicating a favorable safety profile. Nevertheless, reported healing rates and recovery speed vary considerably between studies (17), likely influenced by factors such as disease duration, patient conditions, plug material (18), surgeon experience, acute inflammation, and adequacy of preoperative drainage.

postoperative management included herbal irrigation combined with a standardized regimen of wound care and dressing changes, forming a consistent postoperative protocol that may explain the lower overall recurrence rate compared with previous reports (19). Although the recurrence rate was higher in the AFP group than in the seton group (5.5% vs. 1.5%), this difference was not statistically significant and may be attributable to the limited sample size. While seton therapy has consistently demonstrated low recurrence, achieving comparable reliability with AFP may require stricter case selection and greater operative proficiency.

AFP provide a minimally invasive option for high fistulas, offering less pain and faster recovery while promoting a shift toward sphincter-preserving strategies. The modified plug-plus-loose-seton technique used in this study offers internal opening closure alongside effective drainage, making it suitable for complex tracts.

As a single-center cohort study, selection bias and residual confounding cannot be fully excluded, and the limited sample size may reduce the ability to detect subtle differences in recurrence. Outcomes may also be influenced by surgeon experience and center-specific factors. Further multicenter studies are needed. Future work should focus on optimizing plug materials and postoperative care and comparing this technique with conventional procedures to better define its role in anal fistula management.

## Conclusion

This study examined the clinical utility of an AFP technique that integrates a draining loose seton and an internal-opening management device for high anal fistulas. The findings indicate that this minimally invasive approach can serve as a safe and effective alternative for high-level fistulas, offering clear perioperative advantages such as reduced postoperative pain, faster wound healing, and no additional economic burden. It provides a practical treatment pathway for complex fistulas with internal openings located above the levator ani or the anorectal ring—cases traditionally considered difficult to cure without long-term seton drainage. By preserving sphincter integrity, the technique may help improve healing rates while lowering the risk of recurrence and major complications. Although the long-term efficacy of the plug appears comparable to that of conventional seton therapy, its late recurrence profile requires confirmation in larger cohorts. Overall, the plug-based approach offers clinicians and patients—particularly those prioritizing rapid recovery and quality of life—a well-tolerated therapeutic option with promising clinical applicability. Future studies should aim to define the optimal candidate population and further refine procedural steps to enhance overall outcomes.

## Data Availability

The original contributions presented in the study are included in the article/supplementary material, further inquiries can be directed to the corresponding author.
